# Histogram analysis of diffusion-weighted imaging with a fractional order calculus model in breast cancer: diagnostic performance and associations with prognostic factors

**DOI:** 10.3389/fradi.2025.1664740

**Published:** 2025-12-18

**Authors:** Bo Hu, Caili Tang, Qilan Hu, Xu Yan, Tao Ai

**Affiliations:** 1 Department of Radiology, Yichang Central People’s Hospital, Yichang, China; 2 Department of Radiology, Tongji Hospital, Tongji Medical College, Huazhong University of Science and Technology, Wuhan, China; 3 Scientific Marketing, Siemens Healthineers, Shanghai, China

**Keywords:** breast cancer, diffusion-weighted imaging, fractional order calculus, prognostic biomarkers, histogram analysis, non-Gaussian diffusion

## Abstract

**Objective:**

This study aims to evaluate the diagnostic performance of diffusion-weighted imaging (DWI) with a fractional order calculus (FROC) model for differentiating breast lesions and to explore the associations between FROC/apparent diffusion coefficient (ADC)-derived diffusion metrics and prognostic biomarkers and molecular subtypes in breast cancer.

**Methods:**

This retrospective study included 147 patients with 159 histopathology-confirmed lesions who underwent multi-b DWI using simultaneous multi-slice (SMS) readout-segmented echo-planar imaging (rs-EPI) at 3.0 T. Whole-lesion histograms were computed for mono-exponential ADC and FROC parameters (D, β, μ). The Mann–Whitney *U* test was used to compare the histogram metrics of each diffusion parameter between the benign and malignant groups and between groups with different prognostic biomarkers and molecular subtypes. The Kruskal–Wallis test was used to compare the histogram metrics of each DWI-derived parameter among the different molecular subtypes. The Spearman rank correlation analysis was employed to characterize correlations between diffusion parameters and prognostic biomarkers. The diagnostic performance of each DWI-derived parameter in differentiating breast lesions was assessed using receiver operating characteristic (ROC) analysis.

**Results:**

Interobserver reproducibility was excellent (intra-class correlation coefficient 0.827–0.928). Central tendency histogram metrics (10th, 90th percentiles, mean, median) of ADC and FROC parameters were higher in benign than malignant lesions, whereas skewness (all models) and entropy/kurtosis (ADC, D, μ) were lower in benign lesions (all *p* < 0.05, except β-skewness). The histogram metrics of ADC-median, D_FROC_-mean, and D_FROC_-median showed similar diagnostic performance. The values of ADC-mean, D_FROC_-10%, D_FROC_-mean, D_FROC_-median, β_FROC_-10%, β_FROC_-mean, and β_FROC_-median were significantly lower in the estrogen receptor (ER)-positive group compared with those in the ER-negative group. The tumors with progesterone receptor (PR)-negative status showed significantly higher β_FROC_-10%, β_FROC_-mean, and β_FROC_-median values than those of tumors with PR-positive status. The values of D_FROC_-skewness, β_FROC_-10%, and β_FROC_-mean exhibited significant differences in differentiating the triple-negative and luminal subtypes.

**Conclusions:**

FROC-based histogram analysis yields diagnostic performance comparable to ADC for benign vs. malignant classification, while providing richer associations with ER/PR status, proliferation, and nodal involvement, reflecting microstructural heterogeneity not captured by mono-exponential diffusion.

## Introduction

Breast cancer is the most commonly diagnosed malignancy and a leading cause of cancer-related mortality worldwide (1). Magnetic resonance imaging (MRI) plays an essential role in the detection, characterization, therapy response monitoring, and outcome prediction of breast cancer. However, dynamic contrast-enhanced MRI (DCE-MRI) continues to exhibit a high false-positive rate and low specificity (37%–97%) in identifying breast lesions due to background parenchymal enhancement and overlap of the time-intensity curves between the benign and malignant lesions (2). Previous studies have shown that diffusion-weighted imaging (DWI), as a non-invasive tool, can be an important adjunct imaging sequence to DCE-MRI, improving the diagnostic specificity compared with DCE-MRI alone (3, 4). DWI provides microstructure-sensitive contrast without contrast media with the measurements of the apparent diffusion coefficient (ADC), which can be used in differentiating breast lesions and predicting and monitoring treatment outcomes (5–7).

Breast cancer is a heterogeneous disease comprising multiple biological subtypes with different treatment responses and clinical outcomes (8–11). Therefore, preoperative phenotyping of breast cancer is necessary for optimizing patient-tailored therapy and predicting treatment response. Previous studies have explored the relationship between ADC values and prognostic factors of breast cancer (4, 6, 7, 12). Jeh et al. (13) reported that low ADC-mean values were related to the positive expression of estrogen receptor (ER) and negative expression of human epidermal growth factor receptor 2 (HER2). On the contrary, Kim et al. (5) reported no significant relationship between ADC values and breast cancer prognosis. This disagreement could be explained from the aspect of the basic principles of the different DWI models. The conventional DWI with a mono-exponential model assumes that water motion is homogeneous and follows Gaussian behavior (14, 15). As a matter of fact, the diffusion behavior of water molecules *in vivo* tissue is much more complicated (non-Gaussian distribution) due to the complex tissue microenvironment of tumors, which is not well characterized by the conventional DWI model (16, 17).

In this context, different non-Gaussian diffusion models with new imaging biomarkers, such as intravoxel incoherent motion (IVIM), diffusional kurtosis imaging (DKI), and fractional order calculus (FROC) model, have been proposed to presumably reflect the microstructural heterogeneity and irregularity of tumor cells *in vivo* and the amount of interfaces within cellular tissues (18–22). Different from the IVIM and DKI models, the FROC model is a three-parameter formulation designed to capture complex, scale-dependent diffusion processes: diffusion coefficient D_FROC_ (in square millimeters/second), intravoxel diffusion spatial heterogeneity β_FROC_, and a spatial constant parameter μ_FROC_ (in micrometers) (23). Its parameters potentially reflect tissue organization and heterogeneity beyond ADC. Recently, the FROC model has been proven valuable in differentiating brain tumors, gastrointestinal stromal tumors, bladder urothelial carcinoma, and salivary gland tumors and evaluating therapy responses (23–26). However, the application of the FROC model was not well investigated in breast DWI, particularly for the correlation between the FROC-derived parameters and prognostic biomarkers of breast cancer (27).

The study aims to assess the differential diagnostic performance of FROC-derived histogram features for breast lesions and further evaluate the potential associations between FROC-derived histogram metrics and established prognostic factors [ER, progesterone receptor (PR), HER2, Ki-67] and molecular subtypes, as compared with the conventional mono-exponential DWI model.

## Materials and methods

### Patients

The institutional review board approval was obtained for this retrospective study, and the requirement for informed consent was waived. This study cohort comprised 147 patients who underwent breast MRI for evaluation of suspicious breast lesions between December 2020 and October 2021. The inclusion criteria were as follows: (1) 18 years or older; (2) mass-type lesions with the largest diameter ≥ 1 cm; (3) no history of surgery, chemotherapy, or radiation therapy; and (4) complete clinical data and histopathologic results within 2 weeks after the breast MRI. The exclusion criteria were as follows: (1) non-mass enhancement lesions; (2) lesions with extensive cystic necrosis, making it unable to draw a region of interest (ROI); and (3) poor DWI image quality precluding reliable ROI placement.

### MR imaging protocol

All breast MRI examinations were performed on a 3.0 T scanner (MAGNETOM Skyra, Siemens Healthcare, Erlangen, Germany) with a dedicated 16-channel phased-array bilateral breast coil. The imaging protocol mainly included the following sequences: (1) an axial fast spin-echo T2-weighted imaging (T2WI) sequence with Dixon fat suppression; (2) an axial DWI with simultaneous multi-slice readout-segmented echo-planar imaging (SMS rs-EPI DWI). For the sake of completeness, the diffusion gradient pulse width (*δ* = 19.3 ms) and gradient separation (*Δ* = 40.0 ms) were used in the sequence; and (3) an axial T1-weighted DCE-MRI with time-resolved angiography with stochastic trajectories volume-interpolated breath-hold examination sequence (TWIST-VIBE) technique. The protocol details are shown in Table 1.

**Table 1:** Breast MRI parameters.

Parameters	T2WI	SMS rs-EPI	DCE
TR (ms)	3,700	2,350	5.24
TE (ms)	101	72	2.46
Flip angle (°)	137	180	10
FOV (mm)	320 × 320	280 × 182	320 × 320
Slice thickness (mm)	4.0	5.0	1.5
Bandwidth (Hz/Px)	347	887	780
Acceleration of factor	–	2	–
Readout segments	–	5	–
Fat suppression	SPAIR	SPAIR	Dixon
*b*-values (s/mm^2^)	–	0, 50, 100, 200, 400, 800,1,200, 2,000	–
Averages	1	1 for all	1
Time acquisition	2:06	4:39	5:57

T2WI, T2-weighted image; SMS rs-EPI, simultaneous multislice readout-segmented echo-planar imaging; DCE, dynamic contrast-enhanced; TR, repetition time; TE, echo time; FOV, field of view; SPAIR, spectral attenuated inversion recovery.

### Imaging analysis

The original image data of SMS rs-EPI DWI were post-processed using a non-commercial software (Body Diffusion Laboratory) based on a computing language and interactive environment (BoDiLab, Siemens Healthcare, Erlangen, Germany) (2, 20, 28, 29). Two radiologists with 17 and 5 years of experience in breast MRI interpretation who were blinded to the pathology of each subject independently measured the quantitative ADC and FROC-derived parameters.

The brief post-processing procedures were described as follows: Firstly, a two-dimensional ROI (2D-ROI) was manually drawn on DWI images (*b*-value = 800 s/mm^2^) by selecting the largest cross-sectional slice of each lesion. The cystic components, hemorrhages, calcifications, and vascular structures were avoided. Secondly, a three-dimensional ROI (3D-ROI) of the whole lesion was automatically segmented using the fuzzy C-means (FCM) algorithm built in the software. Thirdly, the voxel-based parametric maps were automatically generated by setting different ranges of *b*-values (0–800 s/mm^2^ for the mono-ADC model; 0–2,000 s/mm^2^ for the FROC model). Lastly, the 3D-ROI was automatically copied onto the parametric maps, and the histogram results of each quantitative parameter were computed and saved for further analysis, including the 10th and 90th percentiles, mean, median, entropy, kurtosis, and skewness. In addition, the signal-to-noise ratio (SNR) was measured on DWI images with *b* = 2,000 s/mm^2^ from randomly selected 30 patients (10 and 20 with benign and malignant lesions, respectively) for the image quality assessment.

According to the FROC model, the voxel intensity within a diffusion-weighted image is given by equation (20):


$$S=S_{0}\exp\left[-D\mu^{2\left(\beta -1\right)}{\left(\gamma G_{d}\delta \right)}^{2\beta }\left(\Delta -\frac{2\beta -1}{2\beta +1}\delta \right)\right]$$

where *S*_0_ is the signal intensity without diffusion-weighting; *G*_d_ is the diffusion gradient amplitude; *δ* is the diffusion gradient pulse width; Δ is the gradient lobe separation (20, 21). D, β, and μ were defined as previously mentioned.

### Histopathologic results

The histopathologic results were retrieved from the electronic medical records of hospital information systems (HIS). The following biomarkers were recorded for each tumor: ER, PR, HER2, Ki-67 labeling index, and axillary lymph node (ALN) status. ER and/or PR expression was considered positive when nuclear immunostaining was ≥1%. HER2 status was scored by immunohistochemistry (IHC) on a 0–3+ scale; tumors with IHC 3+ or 2+ accompanied by gene amplification on fluorescence *in situ* hybridization (FISH) were classified as HER2-positive. High proliferation activity was defined as Ki-67 > 14%.

Breast cancer was subcategorized into four subtypes based on the expression of molecular markers: Luminal A (ER- and/or PR-positive, HER2-negative, Ki-67 < 14%), Luminal B (ER- and/or PR-positive with either HER2 overexpression/amplification or Ki-67 > 14%), triple-negative (ER- and PR-negative, HER2-negative), and HER2-positive type (ER- and PR-negative, HER2-positive) (30, 31). The Luminal A and Luminal B groups were combined into one group (luminal group) for further analysis.

### Statistical analysis

All statistical analyses were performed using IBM SPSS Statistics (version 26.0; IBM Corp., Armonk, NY, USA) and MedCalc statistical software (version 12.0; MedCalc Software Ltd., Ostend, Belgium). Continuous variables were presented as means ± standard deviations (SD), and categorical variables were presented as counts and percentages. The normality of quantitative measurements was evaluated by the Shapiro–Wilk test. An intra-class correlation coefficient (ICC; two-way random-effects model) was applied to assess the inter-reader agreement of the quantitative measurements between two readers. ICCs were interpreted as follows: ≤0.40, poor; 0.40–0.59, fair; 0.60–0.74, good; 0.75–1.00, excellent (32). The Mann–Whitney *U* test was used to compare the histogram results of each diffusion parameter between the benign and malignant groups and the groups with different prognostic factor statuses. The Kruskal–Wallis non-parametric test was used to compare the histogram results of each DWI-derived parameter among the different molecular subtypes. Bonferroni correction was applied to adjust *p*-values for the multiple pairwise comparisons. The Spearman rank correlation analysis was employed to characterize the correlations between diffusion metrics and prognostic factors. The strength of correlation ranged from 0.75 to 1.00 for good, 0.50–0.74 for moderate, 0.25–0.49 for mild, and 0.00–0.24 for poor correlation. Receiver operating characteristic (ROC) analysis with area under the curve (AUC) was applied to assess the diagnostic performance of ADC- and FROC-derived parameters in distinguishing different breast lesions and different molecular subtypes. DeLong's test was used to compare AUCs between parameters. A *p*-value of <0.05 was considered statistically significant.

## Results

### Clinical and histopathologic characteristics

This study included 147 patients (mean age, 48.0 ± 13.6 years; range, 22–68 years) presenting with 159 lesions (50 benign and 109 malignant). Of these patients, 12 have bilateral benign lesions. The lesion size measured on DCE-MRI was 21.4 ± 6.2 mm (14.7–34.3 mm) and 22.1 ± 5.3 mm (14.5–29.0 mm) for the benign and malignant breast lesions, respectively. For the image quality of DWI, the mean SNRs of DWI images with *b* = 2,000 were 22.53 ± 2.90, 56.53 ± 6.56, and 119.56 ± 14.10 for FGT, benign, and malignant lesions, respectively.

Among the benign lesions (*n* = 50), 39 were fibroadenomas (78.0%), 8 adenoses (16.0%), 2 intraductal papillomas (4.0%), and 1 benign phyllodes tumor (2.0%). Among the malignant lesions (*n* = 109), 100 were invasive ductal carcinomas (IDC; 91.7%), 7 mixed ductal carcinomas *in situ* (DCIS) and invasive ductal carcinomas (DCIS/IDC; 6.4%), 1 pure DCIS (0.9%), and 1 encapsulated papillary carcinoma (0.9%).

For cancers (*n* = 109), biomarker profiles were as follows: ER-positive in 66 (60.6%), PR-positive in 69 (63.3%), HER2-positive in 32 (29.4%), and high Ki-67 index in 88 (80.7%). Axillary lymph node metastasis was present in 38 (34.9%). Given the limited number of Luminal B tumors, Luminal A and B tumors were combined into a single luminal category for subtype analyses. Subtype distribution was luminal in 75 (68.8%), HER2-positive in 13 (11.9%), and triple-negative in 21 (19.3%). The details are shown in Table 2.

**Table 2:** Types and distribution of breast lesions within the study population.

Categories	Pathological types	Number (percentage)
Benign (*n* = 50	Fibroadenomas	39 (78.0)
	Adenoses	8 (16.0)
	Papillomas	2 (4.0)
	Benign phyllodes tumor	1 (2.0)
Malignant (*n* = 109	Invasive ductal carcinomas	100 (91.7)
	Mixed ductal carcinomas *in situ* and invasive ductal carcinomas	7 (6.4)
	Ductal carcinoma in situ	1 (0.9)
	Encapsulated papillary carcinomas	1 (0.9)
ER	Positive	66 (60.6)
	Negative	43 (39.4)
PR	Positive	69 (63.3)
	Negative	40 (36.7)
HER2	Positive	32 (29.4)
	Negative	77 (70.6)
Ki-67	Positive	88 (80.7)
	Negative	21 (19.3)
Axillary lymph node	Positive	38 (34.9)
	Negative	71 (65.19)
Molecular subtype	Luminal subtype	75 (68.8)
	HER2-positive subtype	13 (11.9)
	Triple-negative subtype	21 (19.3)

### Diagnostic performance of mono-exponential ADC and FROC-derived parameters

**Figure 1:**
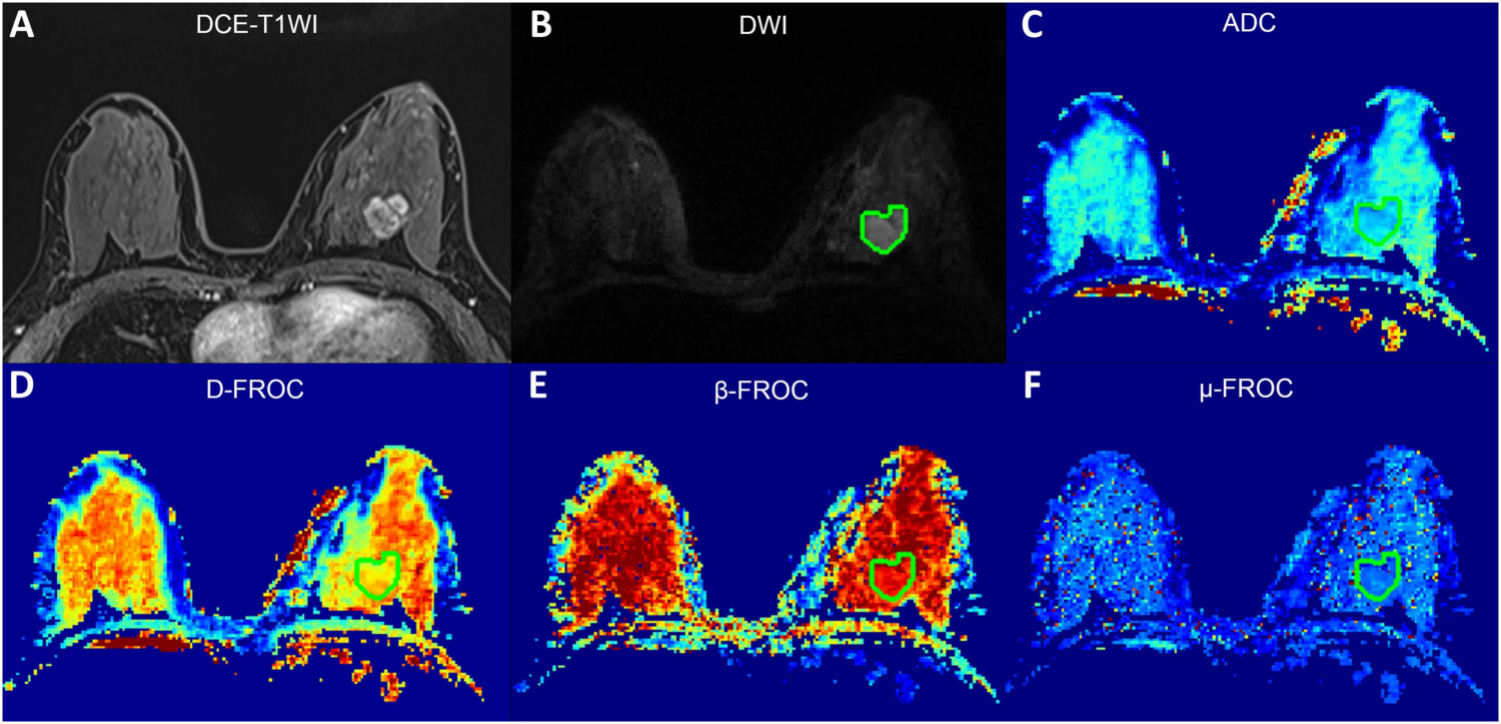
Examples of images for a 27-year-old woman with infiltrating ductal carcinoma in the left breast. Contrast-enhanced T1-weighted image **(A)**, mono-exponential DWI (*b* = 800 s/mm^2^) image **(B)**, ADC image **(C)**, D_FROC_ image **(D)**, β_FROC_ image **(E)**, and μ_FROC_ image **(F)**. The ADC-mean, D_FROC_-mean, β_FROC_-mean, and μ_FROC_ values were 0.994 *×* 10^−3 ^mm^2^/s, 1.088 *×* 10^−3 ^mm^2^/s, 0.787, and 7.075, respectively.

The ICCs for interobserver variability were 0.917 (95% CI, 0.885–0.940), 0.928 (95% CI, 0.900–0.948), 0.833 (95% CI, 0.773–0.878), and 0.827 (95% CI, 0.766–0.874) for ADC, D_FROC_, β_FROC_, and μ_FROC_, respectively, indicating excellent inter-rater agreement. The histogram analysis demonstrated that the differences in ADC, D_FROC_, β_FROC,_ and μ_FROC_ values were statistically different between benign and malignant breast lesions (all *p* < 0.05; Table 3), except for β_FROC_-skewness. The 10% and 90% percentiles, mean, and median values of ADC, D_FROC_, β_FROC_, and μ_FROC_ were significantly higher in benign than those in malignant lesions. Conversely, skewness values of ADC, D_FROC_, β_FROC_, and μ_FROC_ were significantly lower in benign than those in malignant lesions. The entropy and kurtosis values of ADC, D_FROC_, and μ_FROC_ were significantly lower in benign than in malignant lesions. Group-wise distributions for key metrics are illustrated by violin plots (Supplementary Figure S1). The examples of malignant and benign lesions in DCE-MRI, DWI, and diffusion-derived parameter maps are shown in Figure 1 and Supplementary Figure S2.

**Table 3:** Comparison of histogram-derived parameters between benign and malignant lesions and diagnostic performance in differentiating breast lesions.

Histogram parameters	Benign	Malignant	*p*	AUC	Sensitivity (%)	Specificity (%)	Cutoff point
ADC metrics							
ADC-10%	1,182.070 ± 336.607	656.414 ± 126.826	<0.01	0.940	95.41	86.00	848.500
ADC-90%	1,668.982 ± 297.146	1,068.590 ± 171.082	<0.01	0.959	96.33	92.00	1,361.000
ADC-entropy	4.165 ± 0.585	4.376 ± 0.491	0.019	0.616	67.89	58.00	4.200
ADC-kurtosis	3.560 ± 2.483	4.667 ± 2.433	<0.01	0.702	81.65	52.00	2.880
ADC-mean	1,431.755 ± 272.719	843.832 ± 122.565	<0.01	0.979	96.33	94.00	1,028.440
ADC-median	1,439.880 ± 285.282	816.936 ± 121.969	<0.01	0.981	96.33	94.00	1,001.000
ADC-skewness	0.114 ± 0.818	0.880 ± 0.704	<0.01	0.791	75.23	72.00	0.330
D_FROC_ metrics							
D_FROC_-10%	1,255.080 ± 333.550	712.878 ± 148.349	<0.01	0.943	96.33	88.00	933.000
D_FROC_-90%	1,747.994 ± 282.887	1,181.537 ± 180.711	<0.01	0.954	96.33	88.00	1,484.000
D_FROC_-entropy	4.189 ± 0.562	4.540 ± 0.489	<0.01	0.685	70.64	64.00	4.250
D_FROC_-kurtosis	3.538 ± 2.341	4.330 ± 1.960	<0.01	0.686	77.06	58.00	3.030
D_FROC_-mean	1,508.486 ± 256.272	927.381 ± 132.942	<0.01	0.979	96.33	94.00	1,104.52
D_FROC_-median	1,516.810 ± 269.924	901.665 ± 133.399	<0.01	0.979	97.25	92.00	1,098.000
D_FROC_-skewness	0.117 ± 0.794	0.785 ± 0.669	<0.01	0.770	73.39	76.00	0.400
β_FROC_ metrics							
β_FROC_-10%	719.868 ± 176.372	657.139 ± 116.150	<0.01	0.775	96.33	62.00	742.000
β_FROC_-90%	960.416 ± 37.259	895.620 ± 57.903	<0.01	0.811	71.56	92.00	920.500
β_FROC_-entropy	3.275 ± 0.473	3.592 ± 0.433	<0.01	0.697	50.46	90.00	3.650
β_FROC_-kurtosis	17.212 ± 24.568	14.520 ± 13.469	0.037	0.603	88.99	46.00	3.060
β_FROC_-mean	849.692 ± 54.255	772.093 ± 35.107	<0.01	0.880	97.25	72.00	824.500
β_FROC_-median	868.740 ± 54.812	783.211 ± 38.641	<0.01	0.908	84.40	86.00	812.500
β_FROC_-skewness	−2.153 ± 2.345	−1.929 ± 1.675	0.801	0.512	90.83%	28%	−3.790
μ_FROC_ metrics							
μ_FROC_-10%	6,035.350 ± 1,591.250	5,435.192 ± 1,191.744	<0.01	0.765	96.33	66.00	6,317.600
μ_FROC_-90%	9,333.234 ± 1,662.342	8,187.907 ± 1,491.670	<0.01	0.774	66.06	84.00	8,176.00
μ_FROC_-entropy	5.525 ± 0.851	6.066 ± 0.533	<0.01	0.723	88.99	54.00	5.470
μ_FROC_-kurtosis	26.555 ± 22.290	57.157 ± 67.352	<0.01	0.692	66.97	70.00	24.930
μ_FROC_-mean	7,866.594 ± 782.448	6,996.922 ± 702.785	<0.01	0.812	77.98	78.00	7,373.340
μ_FROC_-median	7,329.720 ± 433.453	6,507.206 ± 353.028	<0.01	0.939	88.99	90.00	6,879.000
μ_FROC_-skewness	3.052 ± 3.051	5.236 ± 3.801	<0.01	0.673	66.97	62.00	3.810

Units for ADC and D_FROC_ are 10^−6 ^mm^2^/s. Units for β_FROC_ and μ_FROC_ are 10^−3^.

The ROC analysis showed that ADC-median had the highest discriminative performance (AUC = 0.981; 95% CI, 0.946–0.996). Within the FROC model, the D_FROC_-median and D_FROC_-mean yielded similar AUC values (0.979; 95% CI, 0.943–0.995, and 0.979; 95% CI, 0.942–0.995, respectively). The AUC of ADC-median was not significantly different from those with D_FROC_-mean and D_FROC_-median (Delong *p* = 0.477 and 0.343). In multivariable models that combined all histogram metrics reaching statistical significance within each framework, overall discrimination was excellent for both ADC and FROC (AUCs 0.981 and 0.992, respectively), with no statistically significant difference between the two (DeLong test, *p* = 0.182).

### Correlation analysis of mono-exponential ADC and FROC-derived parameters and prognostic factors and molecular subtypes

ER-negative tumors exhibited significantly higher ADC-mean values and lower ADC-median values than the ER-positive tumors (both *p* < 0.05; Table 4). The patients with ALN metastasis had higher ADC-entropy than node-negative patients (*p* = 0.019). No significant differences in the histogram metrics were observed across PR status, HER2 status, and Ki-67 categories, and no subtype-level differences were detected (all *p* > 0.05).

**Table 4:** Association of ADC histograms (× 10^−6^ mm^2^/s) with prognostic factors and subtype in histogram analysis.

Prognostic factors	*n* (%)	10%	90%	Mean	Median	Entropy	Kurtosis	Skewness
ER								
Negative	43 (39.45)	692.232 ± 115.856	1,094.291 ± 171.935	874.645 ± 114.089	4.360 ± 0.524	4.360 ± 0.524	4.706 ± 1.949	0.945 ± 0.666
Positive	66 (60.55)	633.079 ± 129.038	1,051.846 ± 169.731	822.549 ± 124.347	4.385 ± 0.472	4.385 ± 0.472	4.640 ± 2.717	0.838 ± 0.730
*p*		0.130	0.194	**0.014**	**0.014**	0.913	0.171	0.486
PR								
Negative	40 (36.70)	672.273 ± 124.138	1,072.750 ± 180.534	855.694 ± 122.232	830.000 ± 116.290	4.340 ± 0.533	4.683 ± 1.975	0.944 ± 0.633
Positive	69 (63.30)	647.222 ± 128.353	1,066.178 ± 166.665	835.802 ± 123.054	809.362 ± 125.350	4.396 ± 0.467	4.656 ± 2.677	0.844 ± 0.744
*p*		0.338	0.942	0.333	0.297	0.593	0.305	0.414
HER2								
Negative	77 (70.64)	649.246 ± 121.498	1,056.961 ± 163.148	832.499 ± 113.321	804.266 ± 111.673	4.352 ± 0.515	4.838 ± 2.608	0.922 ± 0.753
Positive	32 (29.36)	673.666 ± 139.327	1,096.572 ± 188.600	868.615 ± 141.072	847.422 ± 141.046	4.433 ± 0.429	4.252 ± 1.922	0.779 ± 0.568
*p*		0.383	0.466	0.183	0.138	0.700	0.331	0.328
Ki-67								
Low expression	21 (19.27)	648.886 ± 136.257	1,090.067 ± 206.968	847.449 ± 160.716	821.571 ± 166.395	4.399 ± 0.463	4.343 ± 1.902	0.878 ± 0.689
High expression	88 (80.73)	658.211 ± 125.229	1,063.465 ± 162.323	842.065 ± 112.715	815.830 ± 109.981	4.370 ± 0.499	4.743 ± 2.547	0.881 ± 0.711
*p*		0.526	0.601	0.741	0.797	0.607	0.812	0.812
Axillary lymph node								
Non-metastasis	71 (65.14)	674.254 ± 128.457	1,070.525 ± 187.255	852.618 ± 133.526	824.972 ± 133.634	4.297 ± 0.513	4.852 ± 2.522	0.983 ± 0.748
Metastasis	38 (34.86)	623.084 ± 118. 236	1,064.974 ± 138.113	825.322 ± 98.076	801.921 ± 96.345	4.523 ± 0.413	4.318 ± 2.249	0.690 ± 0.576
*p*		0.074	0.821	0.285	0.421	**0.019**	0.255	0.062
Subtype								
HER2-positive	21 (19.27)	704.519 ± 111.962	1,084.771 ± 191.975	871.343 ± 123.412	835.310 ± 117.778	4.245 ± 0.591	5.019 ± 1.779	1.173 ± 0.571
Luminal	75 (68.81)	642.305 ± 127.978	1,061.095 ± 165.499	832.614 ± 121.971	807.960 ± 124.195	4.402 ± 0.472	4.601 ± 2.619	0.814 ± 0.731
Triple-negative	13 (11.93)	660.108 ± 132.520	1,085.692 ± 178.790	857.986 ± 125.503	839.039 ± 118.441	4.433 ± 0.421	4.473 ± 2.330	0.792 ± 0.659
*p*		0.143	0.880	0.347	0.415	0.676	0.097	0.070

ER, estrogen receptor; PR, progesterone receptor; HER2, human epidermal growth factor receptor 2.

**Bold values: *p*:**  < 0.05.

ER-negative tumors showed significantly higher D_FROC_-10%, D_FROC_-mean, and D_FROC_-median values than those in ER-positive tumors (*p* = 0.027, 0.031, 0.041, respectively; Table 5). Patients with ALN metastasis had significantly higher D_FROC_-entropy values and lower D_FROC_-skewness values than those of patients without ALN metastasis (*p* = 0.014 and 0.021). No significant differences were found by PR, HER2, and Ki-67 groups. Across molecular subtypes, D_FROC_-skewness differed overall (Kruskal–Wallis *p* = 0.032); in pairwise comparisons, D_FROC_-skewness differed significantly between the luminal and the triple-negative subtypes (*p* = 0.044) (Supplementary Figure S3). The HER2-positive subtype tended to show higher D_FROC_-skewness values than both luminal and triple-negative subtypes.

**Table 5:** Association of D_FROC_ histograms (× 10^−6 ^mm^2^/s) with prognostic factors and subtype in histogram analysis.

Prognostic factors	*n* (%)	10%	90%	Mean	Median	Entropy	Kurtosis	Skewness
ER								
Negative	43 (39.45)	747.947 ± 128.957	1,204.351 ± 181.084	955.875 ± 122.054	926.802 ± 116.089	4.531 ± 0.513	4.306 ± 1.598	0.855 ± 0.631
Positive	66 (60.55)	690.030 ± 156.451	1,166.673 ± 180.286	908.818 ± 137.301	885.288 ± 142.019	4.545 ± 0.477	4.346 ± 2.172	0.739 ± 0.693
*p*		**0** **.** **027**	0.193	**0** **.** **031**	**0** **.** **041**	0.931	0.385	0.330
PR								
Negative	40 (36.70)	728.695 ± 139.975	1,184.433 ± 192.486	937.634 ± 130.273	910.238 ± 123.777	4.506 ± 0.527	4.290 ± 1.662	0.872 ± 0.587
Positive	69 (63.30)	703.709 ± 153.242	1,179.858 ± 174.954	921.437 ± 135.051	896.696 ± 139.313	4.559 ± 0.469	4.353 ± 2.122	0.734 ± 0.711
*p*		0.362	0.819	0.414	0.384	0.589	0.580	0.346
HER2								
Negative	77 (70.64)	705.634 ± 140.335	1,172.082 ± 177.898	917.568 ± 124.668	889.779 ± 123.270	4.527 ± 0.519	4.460 ± 2.077	0.821 ± 0.709
Positive	32 (29.36)	730.309 ± 167.168	1,204.288 ± 188.221	950.994 ± 150.514	930.266 ± 153.453	4.571 ± 0.415	4.018 ± 1.619	0.697 ± 0.560
*p*		0.295	0.523	0.250	0.228	0.765	0.293	0.278
Ki-67								
Low expression	21 (19.27)	716.581 ± 153.665	1,208.105 ± 216.110	942.365 ± 175.389	918.810 ± 187.222	4.556 ± 0.457	4.129 ± 1.704	0.770 ± 0.684
High expression	88 (80.73)	711.994 ± 147.943	1,175.197 ± 172.022	923.806 ± 121.659	897.574 ± 118.088	4.536 ± 0.499	4.378 ± 2.020	0.788 ± 0.669
*p*		0.645	0.604	0.945	0.936	0.690	0.902	0.957
Axillary lymph node								
Non-metastasis	71 (65.14)	734.458 ± 143.195	1,181.355 ± 196.012	937.101 ± 143.577	908.430 ± 145.117	4.458 ± 0.508	4.510 ± 2.044	0.898 ± 0.689
Metastasis	38 (34.86)	672.558 ± 151.266	1,181.876 ± 150.445	909.220 ± 109.853	889.026 ± 108.857	4.693 ± 0.416	3.993 ± 1.762	0.572 ± 0.579
*p*		0.107	0.881	0.405	0.668	**0** **.** **014**	0.176	**0** **.** **021**
Subtype								
HER2-positive	21 (19.27)	762.586 ± 118.614	1,198.433 ± 213.653	952.067 ± 135.027	912.214 ± 126.094	4.415 ± 0.577	4.568 ± 1.473	1.099 ± 0.521
Luminal	75 (68.81)	699.208 ± 152.596	1,173.916 ± 174.456	917.747 ± 133.491	894.680 ± 137.621	4.561 ± 0.473	4.315 ± 2.091	0.717 ± 0.696
Triple-negative	13 (11.93)	711.446 ± 159.778	1,198.208 ± 170.246	943.084 ± 129.894	924.923 ± 125.611	4.618 ± 0.429	4.032 ± 1.929	0.669 ± 0.607
*p*		0.234	0.768	0.410	0.509	0.547	0.159	**0** **.** **032**

ER, estrogen receptor; PR, progesterone receptor; HER2, human epidermal growth factor receptor 2.

**Bold values: *p*:**  < 0.05.

ER-negative and PR-negative tumors demonstrated significantly higher β_FROC_-10%, β_FROC_-mean, and β_FROC_-median than those in ER-positive tumors (all *p* < 0.05; Table 6). High Ki-67 tumors showed significantly higher β_FROC_-10% and β_FROC_-mean and lower β_FROC_-entropy values than those in low Ki-67 tumors (*p* = 0.001, 0.007, and 0.038). β_FROC_-10% and β_FROC_-mean differed across molecular subtypes (*p* = 0.002 and 0.016); pairwise comparisons showed both β_FROC_-10% and β_FROC_-mean were significantly higher in triple-negative than those in luminal tumors (*p* = 0.001 and 0.012). β_FROC_-10% and β_FROC_-mean also tended to be higher in HER2-positive than those in the other groups.

**Table 6:** Association of β_FROC_ histograms (× 10^−3^) with prognostic factors and subtype in histogram analysis.

Prognostic factors	*n* (%)	10%	90%	Mean	Median	Entropy	Kurtosis	Skewness
ER								
Negative	43 (39.45)	679.472 ± 96.225	892.574 ± 54.868	779.515 ± 39.228	788.058 ± 41.756	3.519 ± 0.422	15.078 ± 13.898	−1.871 ± 1.796
Positive	66 (60.55)	642.588 ± 126.047	897.605 ± 60.128	767.258 ± 31.517	780.053 ± 36.451	3.639 ± 0.436	14.155 ± 13.277	−1.968 ± 1.605
*p*		**0.001**	0.814	**0.002**	**0.020**	0.159	0.892	0.862
PR								
Negative	40 (36.70)	676.808 ± 99.630	895.298 ± 54.699	779.004 ± 41.206	788.713 ± 43.184	3.541 ± 0.434	15.273 ± 14.254	−1.926 ± 1.818
Positive	69 (63.30)	645.736 ± 123.993	895.807 ± 60.075	768.087 ± 30.649	780.022 ± 35.691	3.620 ± 0.433	14.082 ± 13.079	−1.931 ± 1.600
*p*		**0.004**	0.751	**0.007**	**0.014**	0.414	0.792	0.870
HER2								
Negative	77 (70.64)	664.861 ± 96.157	898.878 ± 59.243	774.052 ± 31.907	783.942 ± 37.852	3.588 ± 0.432	15.068 ± 14.315	−1.985 ± 1.681
Positive	32 (29.36)	638.556 ± 154.364	887.781 ± 54.648	767.380 ± 42.019	781.453 ± 41.047	3.601 ± 0.441	13.197 ± 11.273	−1.795 ± 1.680
p		0.709	0.709	0.714	0.803	0.931	0.637	0.754
Ki-67								
Low expression	21 (19.27)	635.762 ± 61.153	902.495 ± 62.770	760.157 ± 22.914	773.429 ± 23.300	3.773 ± 0.382	15.082 ± 12.453	−2.250 ± 1.402
High expression	88 (80.73)	662.240 ± 125.503	893.980 ± 56.940	774.943 ± 36.969	785.546 ± 41.234	3.548 ± 0.435	14.385 ± 13.764	−1.853 ± 1.733
*p*		**0.001**	0.539	**0.007**	0.088	**0.038**	0.544	0.381
Axillary lymph node								
Non-metastasis	71 (65.14)	669.996 ± 81.360	893.765 ± 59.492	774.636 ± 32.305	784.859 ± 34.308	3.549 ± 0.425	14.136 ± 12.543	−1.900 ± 1.689
Metastasis	38 (34.86)	633.116 ± 161.077	899.087 ± 55.423	767.342 ± 39.845	780.132 ± 46.006	3.670 ± 0.443	15.235 ± 15.201	−1.985 ± 1.672
*p*		0.133	0.472	0.484	0.593	0.133	0.746	0.849
Subtype								
HER2-positive	21 (19.27)	701.643 ± 54.864	890.071 ± 48.497	784.957 ± 38.297	789.833 ± 42.171	3.508 ± 0.359	18.937 ± 15.471	−2.278 ± 1.829
Luminal	75 (68.81)	647.845 ± 120.125	897.868 ± 59.006	769.526 ± 30.806	781.427 ± 35.192	3.632 ± 0.435	13.766 ± 12.768	−1.909 ± 1.585
Triple-negative	13 (11.93)	638.862 ± 151.820	883.536 ± 67.781	766.124 ± 49.259	782.808 ± 52.350	3.489 ± 0.520	11.724 ± 13.505	−1.485 ± 1.942
*p*		**0.002**	0.690	**0.016**	0.127	0.368	0.199	0.338

ER, estrogen receptor; PR, progesterone receptor; HER2, human epidermal growth factor receptor 2.

**Bold values: *p*:**  < 0.05.

Among the histogram μ_FROC_ values, only μ_FROC_-entropy differed by Ki-67 status (*p* = 0.047; Table 7). No significant differences were observed among molecular subtypes (all *p* > 0.05).

**Table 7:** Association of μ_FROC_ histograms (× 10^−3^) with prognostic factors and subtype in histogram analysis.

Prognostic factors	*n* (%)	10%	90%	Mean	Median	Entropy	Kurtosis	Skewness
ER								
Negative	43 (39.45)	5,549.998 ± 1,083.598	8,033.509 ± 1,428.431	6,943.614 ± 686.883	6,538.012 ± 324.404	6.115 ± 0.451	62.524 ± 67.644	5.568 ± 3.862
Positive	66 (60.55)	5,360.394 ± 1,259.643	8,288.500 ± 1,533.815	7,031.652 ± 716.017	6,487.136 ± 371.535	6.035 ± 0.582	53.660 ± 67.448	5.019 ± 3.775
*p*		0.359	0.183	0.244	0.438	0.382	0.757	0.535
PR								
Negative	40 (36.70)	5,496.735 ± 1,130.546	1,072.750 ± 180.534	855.694 ± 122.232	6,629.700 ± 339.738	6.098 ± 0.453	4.683 ± 1.975	0.944 ± 0.633
Positive	69 (63.30)	647.222 ± 128.353	1,066.178 ± 166.665	835.802 ± 123.054	6,494.167 ± 362.316	6.048 ± 0.577	4.656 ± 2.677	0.843 ± 0.744
*p*		0.660	0.845	0.615	0.635	0.542	0.263	0.501
HER2								
Negative	77 (70.64)	5,417.703 ± 1,143.764	8,239.366 ± 1,554.594	7,004.596 ± 733.138	6,510.955 ± 350.751	6.072 ± 0.554	54.346 ± 71.264	4.889 ± 3.890
Positive	32 (29.36)	5,477.275 ± 1,318.408	8,064.109 ± 1,343.330	6,978.454 ± 634.421	6,498.1875 ± 363.947	6.054 ± 0.486	63.918 ± 57.330	6.069 ± 3.480
*p*		0.644	0.497	0.821	0.997	0.765	0.147	0.096
Ki-67								
Low expression	21 (19.27)	5,519.600 ± 869.690	8,432.948 ± 1,456.971	7,157.264 ± 530.628	6,542.429 ± 322.524	5.909 ± 0.596	41.091 ± 31.222	4.699 ± 2.722
High expression	88 (80.73)	5,415.049 ± 1,259.788	8,129.432 ± 1,502.073	6,958.658 ± 735.331	6,498.801 ± 361.147	6.104 ± 0.514	60.990 ± 73.007	5.364 ± 4.019
*p*		0.975	0.272	0.072	0.664	0.142	0.684	0.656
Axillary lymph node								
Non-metastasis	71 (65.14)	5,501.828 ± 990.275	8,122.024 ± 1,341.590	7,001.556 ± 684.535	6,615.782 ± 323.240	5.993 ± 0.566	53.388 ± 58.118	4.962 ± 3.822
Metastasis	38 (34.86)	5,310.687 ± 1,505.286	8,311.005 ± 1,750.939	6,988.263 ± 745.010	6,491.184 ± 407.067	6.203 ± 0.440	64.197 ± 82.297	5.748 ± 3.760
*p*		0.670	0.480	0.643	0.770	**0** **.** **047**	0.347	0.387
Subtype								
HER2-positive	21 (19.27)	5,581.229 ± 1,158.545	8,103.095 ± 1,368.475	6,987.416 ± 787.937	6,541.167 ± 358.494	6.132 ± 0.417	59.969 ± 73.611	5.261 ± 3.995
Luminal	75 (68.81)	5,394.547 ± 1,202.006	8,202.879 ± 1,464.157	7,006.409 ± 679.006	6,490.820 ± 358.376	6.070 ± 0.572	57.399 ± 67.695	5.318 ± 3.777
Triple-negative	13 (11.93)	5,433.777 ± 1,260.108	8,238.538 ± 1,915.182	6,957.543 ± 751.588	6,546.885 ± 331.743	5.939 ± 0.475	51.217 ± 59.026	4.723 ± 3.893
*p*		0.647	0.668	0.750	0.662	0.478	0.583	0.774

ER, estrogen receptor; PR, progesterone receptor; HER2, human epidermal growth factor receptor 2.

**Bold value: *p*:**  < 0.05.

### Spearman correlation analysis

**Figure 2:**
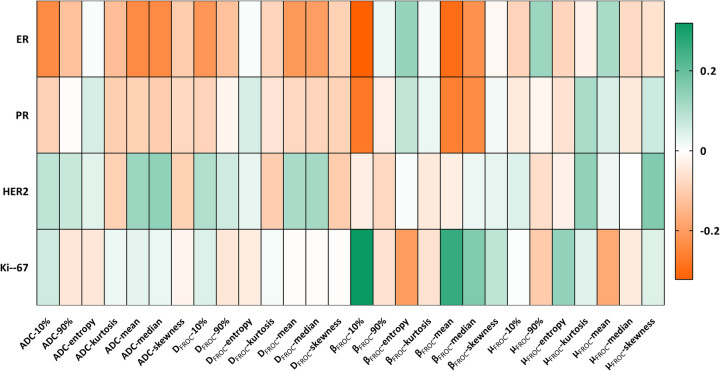
Spearman correlations between DWI parameters and prognostic factors are charted in a heat map. Colored entries indicate Spearman correlation with positive (orange) or negative (green).

Correlation analyses showed predominantly weak-to-moderate negative associations between receptor expression and diffusion metrics (Figure 2). ER expression correlated negatively with ADC-10% (*r* = −0.238, *p* = 0.013), ADC-mean (*r* = −0.237, *p* = 0.013), and ADC-median (*r* = −0.237, *p* = −0.123). ER was also negatively correlated with D_FROC_-10% (*r* = −0.213, *p* = 0.026), D_FROC_-mean (*r* = −0.208, *p* = 0.030), D_FROC_-median (*r* = −0.197, *p* = 0.040), β_FROC_-10% (*r* = −0.322, *p* = 0.001), β_FROC_-mean (*r* = −0.295, *p* = 0.002), and β_FROC_-median (*r* = −0.224, *p* = 0.019).

PR expression correlated negatively with β_FROC_-10% (*r* = −0.273, *p* = 0.004), β_FROC_-mean (*r* = −0.258, *p* = 0.007), and β_FROC_-median (*r* = −0.236, *p* = 0.014). Ki-67 was negatively correlated with β_FROC_-entropy (*r* = −0.200, *p* = 0.037) and β_FROC_-mean (*r* = 0.261, *p* = 0.006) and positively correlated with β_FROC_-10% (*r* = 0.319, *p* = 0.001). No μ_FROC_ metric correlated significantly with prognostic factors, and no diffusion metric showed a significant correlation with HER2 status.

### Diagnostic performance for molecular subtype discrimination

**Figure 3:**
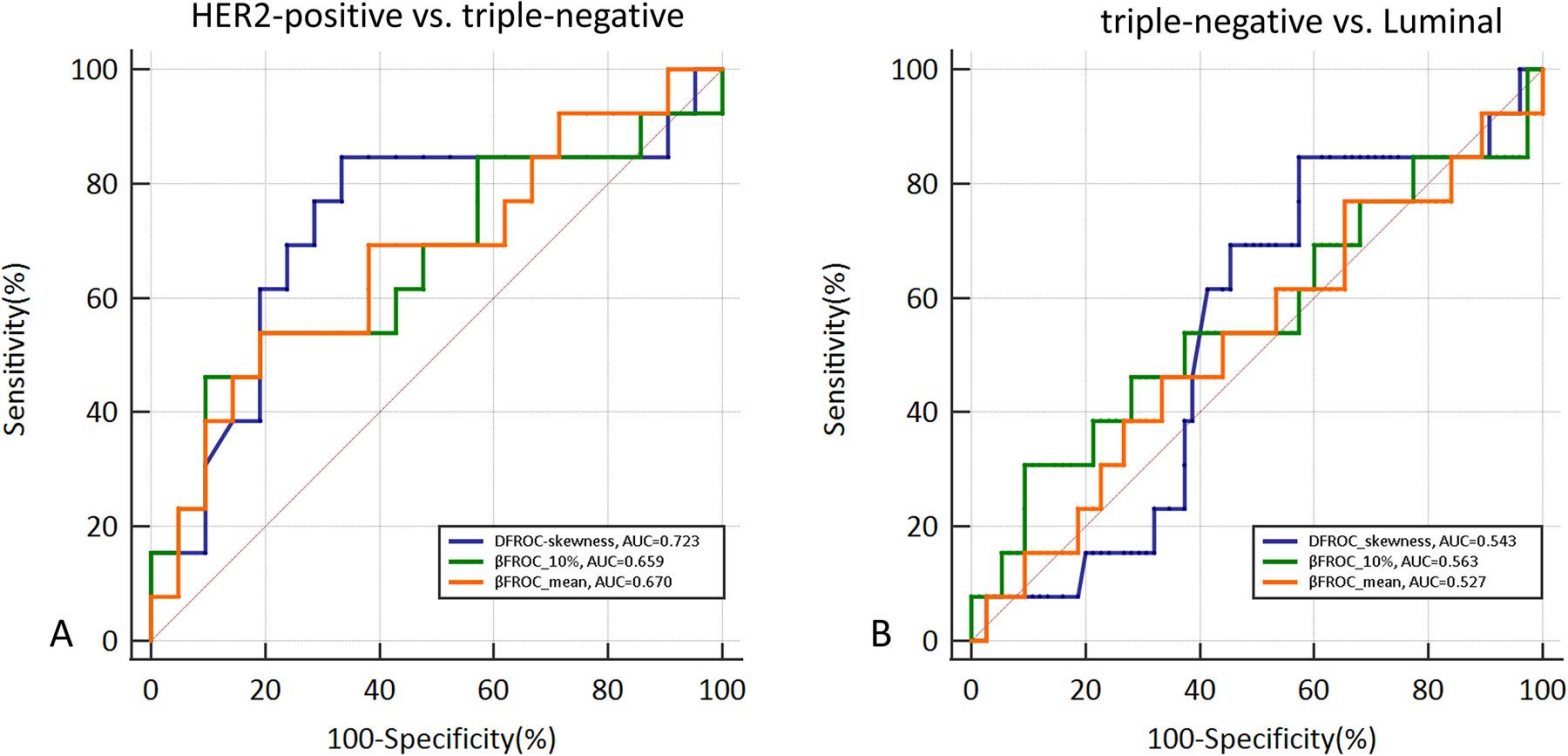
ROC curves of D_FROC_-skewness, β_FROC_-10% and β_FROC_-mean in distinguishing HER2-positive and triple-negative **(A)**, triple-negative and Luminal **(B)**.

Figure 3 shows the ROC curves for D_FROC_-skewness, β_FROC_-10%, and β_FROC_-mean in two pairwise classification tasks: HER2-positive vs. triple-negative and triple-negative vs. luminal. The AUC, sensitivity, and specificity were 0.723, 84.62%, and 66.67% for D_FROC_-skewness; 0.659, 46.15%, and 90.48% for β_FROC_-10%; and 0.670, 53.85%, and 80.95% for β_FROC_-mean in distinguishing HER2-positive and triple-negative. The AUC, sensitivity, and specificity were 0.543, 84.62%, and 42.67% for D_FROC_-skewness; 0.563, 30.77%, and 90.67% for β_FROC_-10%; 0.586, 30.77%, and 95.24% for β_FROC_-mean in distinguishing triple-negative and luminal.

## Discussion

In this study, whole-lesion histogram analysis of quantitative DWI with mono-exponential and FROC models was performed to differentiate breast lesions and to explore associations between diffusion metrics and prognostic biomarkers in breast cancer. The results demonstrated that the diagnostic performance of D_FROC_ was comparable to that of ADC for benign–malignant discrimination. However, the histogram features derived from FROC models showed broader and stronger associations with prognostic biomarkers and molecular subtypes than did ADC metrics, features of breast cancer compared with the mono-ADC values, suggesting that non-Gaussian parameters capture complementary aspects of tumor microstructure and heterogeneity beyond conventional diffusion (21). These findings may help guide therapy decisions and predict breast cancer prognosis.

The FROC model is a non-Gaussian model proposed for better characterization of the complexity of biological tissue with three diffusion parameters (D_FROC_, β_FROC_, μ_FROC_). It is important to note that when data are acquired by varying the diffusion gradient strength at a fixed diffusion time, the signal decay fitted by the FROC model reflects a pseudo-superdiffusion mechanism. This contrast arises from the interplay between water diffusion multi-compartmentalization and local magnetic field inhomogeneities generated by magnetic susceptibility differences (Δ*χ*) at the interfaces between different tissue compartments (33, 34). D_FROC_ was higher in benign than that in malignant breast lesions, which was aligned with a previous study (27). In our study, D_FROC_ had similar diagnostic performance compared with mono-ADC values. Beyond averaged estimates, the distributional shape of voxel-wise metrics carried clinically relevant information. In our cohort, 10th/90th percentiles, mean, and median of ADC and FROC parameters were consistently higher in benign than those in malignant lesions, whereas skewness (all models) and entropy/kurtosis (ADC, D_FROC_, μ_FROC_) were lower in benign. This pattern suggests that benign lesions tend to have more homogeneous diffusivity, with fewer extreme voxels. In contrast, malignant lesions exhibit heavier tails and greater disorder, consistent with intermixed regions of high cellularity, stromal remodeling, edema, and necrosis.

Recent clinical applications of SMS rs-EPI with advanced non-Gaussian modeling, including FROC, further support its diagnostic feasibility in the breast (35). Our study was based on the SMS rs-EPI, which produced superior image quality and lesion characterization than ss-EPI (28). Differences between our results and earlier ss-EPI–based FROC studies likely reflect these acquisition advantages and cohort size/mixture effects, both of which influence histogram-based AUCs. Moreover, the pathological composition was relatively simple in our study. The malignant lesions were mostly invasive ductal carcinomas (*n* = 100, 62.89%), and the benign lesions were fibroadenomas (*n* = 39, 24.53%).

In addition, both β_FROC_ and μ_FROC_ are spatial parameters that reflect the underlying tissue structural complexity. β_FROC_ represents the fractional order derivative in space, and μ_FROC_ is considered a measure of diffusion mean free length. In the context of pseudo-superdiffusion, the β_FROC_ parameter has been shown to be particularly sensitive to local magnetic susceptibility differences (Δ*χ*) (33, 34). Several studies reported that β_FROC_ values and μ_FROC_ values were inversely associated with intravoxel tissue heterogeneity (20, 21, 23). In our cohort, the mean values of β_FROC_ and μ_FROC_ were higher in benign lesions than those in malignant lesions, indicating that malignant tumors harbor broader voxel-wise heterogeneity and heavier-tailed distributions, consistent with admixtures of densely cellular regions, edema/necrosis, and stromal remodeling.

A critical consideration when applying the FROC model in the breast is the potential confounding effect of magnetic susceptibility. Breast tissue is characterized by a significant Δ*χ* at the interfaces between adipose tissue and water-rich glandular or tumor tissue. The internal magnetic fields (G_int) generated by this Δ*χ* can couple with the applied diffusion gradients, contributing to the signal decay in a way that mimics a superdiffusion process, thereby influencing the estimated β_FROC_. Therefore, while our results show strong correlations between β_FROC_ and prognostic factors, the biological interpretation is complex. The observed associations might not solely reflect tissue microstructure (heterogeneity) but could also be modulated by susceptibility-related effects linked to angiogenesis, inflammatory infiltration, or other pathological features that alter the local magnetic landscape (33).

We observed consistent (generally mild-to-moderate) negative correlations between receptor expression and non-Gaussian metrics, with β_FROC_ features showing the broadest associations across ER, PR, and Ki-67. The biological directionality is nuanced. ER-/PR-negative cancers typically show higher microvessel density and more aggressive phenotypes than hormone receptor–positive tumors, which may alter extracellular water mobility and the balance between restricted and hindered components (36, 37). The ER- and PR–PR-negative groups showed significantly higher β_FROC_-mean values, indicating the low heterogeneity in the group. Meanwhile, Ki-67 reflects proliferative activity and tumor aggressiveness; meta-analyses and multicenter data indicate that ADC-Ki-67 correlations are weak or inconsistent, underscoring the value of models sensitive to heterogeneity beyond mean diffusivity (38, 39). The low expression Ki-67 group showed higher ADC- and D_FROC_-mean values, demonstrating the low cell density in the low expression group. Moreover, the β_FROC_-mean was higher in the high Ki-67 expression group, which was inconsistent with the high heterogeneity in the group. In this context, the stronger and more pervasive associations of β_FROC_ with ER/PR/Ki-67 suggest that anomalous diffusion metrics may better index voxel-scale disorder and microarchitectural dispersion than ADC alone.

HER2 expression induced angiogenesis and aggressiveness (36). The HER2 overexpression group had higher ADC- and D_FROC_-mean values, which could be explained by higher blood flow in the tumor tissue (40). In the correlation analysis, the ADC and D_FROC_ histogram metrics showed a significant negative correlation with ER expression. The β_FROC_ histogram metrics were significantly correlated with ER, PR, and Ki-67 status. However, we did not detect robust correlations between diffusion metrics and HER2 status in this cohort, indicating that perfusion/vascular effects may be less directly captured by diffusion-weighted metrics than by DCE-MRI or perfusion imaging.

Regarding molecular subtypes, the luminal subtype was typically associated with a more favorable prognosis compared with the triple-negative and HER2-positive subtypes. The triple-negative tumors lacking expression of ER, PR, and HER2 were characterized by more invasive characteristics, a high recurrence rate, and poor prognosis (41). The luminal subtypes have relatively higher cellularity and vascular density compared with the triple-negative subtype. In our study, D_FROC_-skewness showed the highest AUC value in distinguishing between HER2-positive and triple-negative tumors, and β_FROC_-mean produced the highest AUC value in distinguishing between triple-negative and luminal tumors. Although the resulting AUCs were modest (approximately 0.527–0.723) and therefore insufficient for standalone subtype classification, these orthogonal signals—with percentiles indexing the burden of abnormal voxels and shape metrics capturing distributional asymmetry/disorder—are suited for multivariable integration (e.g., radiomic features) and decision curve–oriented model building, where complementary contributions may yield clinically meaningful net benefit.

There were several limitations in this study. First, this study was conducted in a single institution, although the data were reliable. We need to validate the results in multicenter research in the future. Second, a fundamental limitation of our study is the inherent coupling between microstructural and magnetic susceptibility effects in the FROC parameters derived from our acquisition scheme. We could not disentangle the contribution of genuine water diffusion heterogeneity from that induced by Δ*χ*. Future studies employing bipolar diffusion gradients or acquisitions at multiple magnetic field strengths could help separate these effects. Third, we did not obtain many studies on the FROC model in breast cancer. This might influence the explanation of the derived parameters. Fourth, the 2D-ROI was manually drawn on DWI maps (*b* = 800 s/mm2) and used to produce a 3D-ROI automatically. The 3D-ROI was then copied to the derived parameter maps. Careful comparison of the DWI maps and derived parameter maps of the FROC model is necessary when setting an ROI for each lesion in the future study.

## Conclusions

Whole-lesion histogram analysis of FROC-modeled DWI provides diagnostic performance comparable to ADC for distinguishing benign from malignant breast lesions, while yielding richer associations with ER/PR status and proliferative activity that likely reflect microstructural heterogeneity not captured by mono-exponential decay. These non-Gaussian metrics, particularly β_FROC_ features, represent promising adjunct biomarkers that can potentially provide additional imaging biomarkers to guide therapy decisions and predict breast cancer prognosis.

## Data Availability

The raw data supporting the conclusions of this article will be made available by the authors, without undue reservation.
