# Supplementary material for: Histogram analysis of diffusion-weighted imaging with a fractional order calculus model in breast cancer: diagnostic performance and associations with prognostic factors
Source: Front Radiol. 2025 Dec 18;5:1664740. doi: 10.3389/fradi.2025.1664740 (PMC12756068; doi:10.3389/fradi.2025.1664740)
Supplement: Supplementary file 1 [file Datasheet1.pdf]

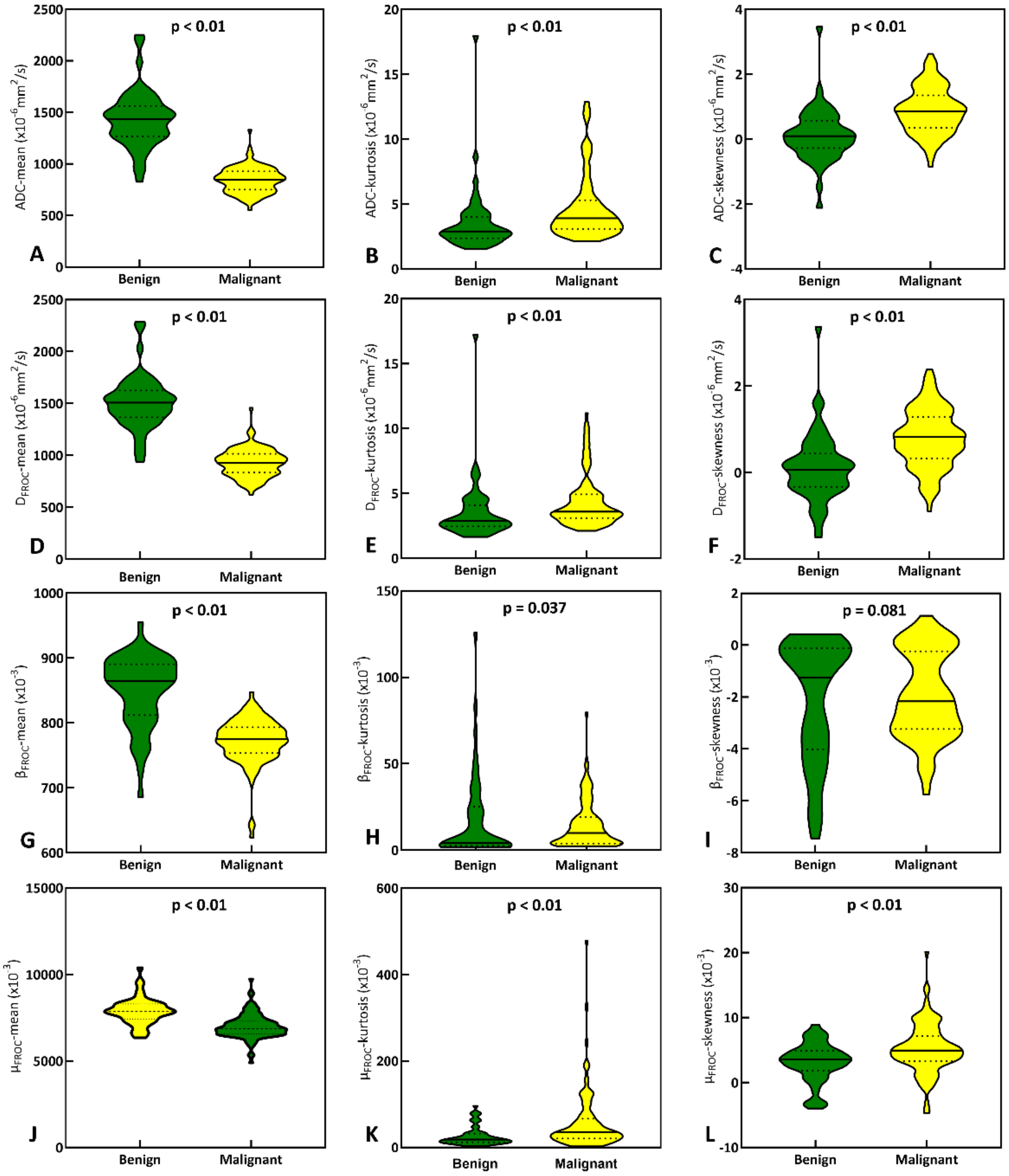

Supplementary Figure 1. Violin plots show the mean, kurtosis, and skewness of all derived parameters for benign and malignant lesions. ADC-mean (A), ADC-kurtosis (B), ADC-skewness (C), D_{FROC}-mean (D), D_{FROC}-kurtosis (E), D_{FROC}-skewness (F), β_{FROC}-mean (G), β_{FROC}-kurtosis (H), β_{FROC}-skewness (I),μ_{FROC}-mean (J), μ_{FROC}-kurtosis (K), μ_{FROC}-skewness (L). The solid line in the box represents the median value. The top and bottom dotted line in the box represents the 25th and 75th percentile values, respectively.

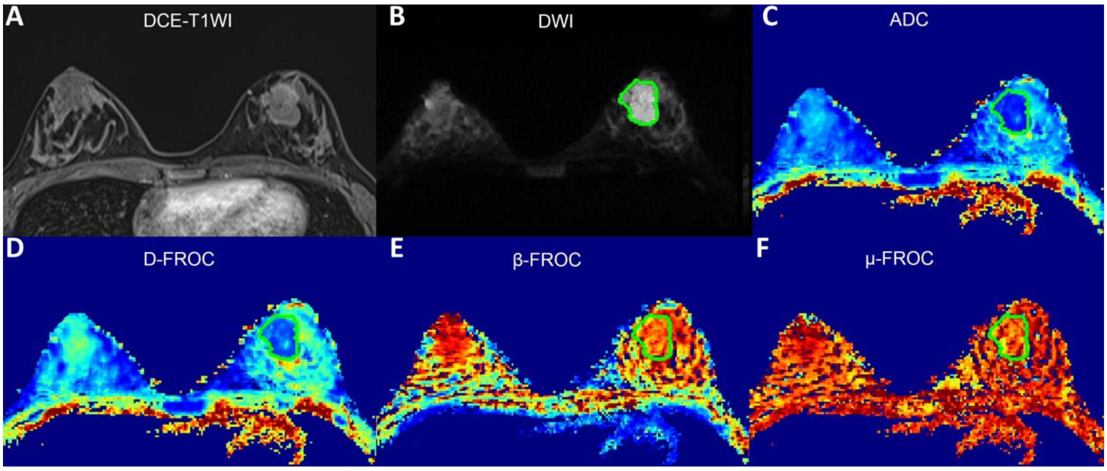

Supplementary Figure 2. Examples of images for a 29-years woman with fibroadenomas in the left breast. Contrast enhanced T1-weighted image (A), mono-exponential DWI (b = 800 s/mm^2) image (B), ADC image (C), D_{FROC} image (D), β_{FROC} image (E), and μ_{FROC} image (F). The ADC-mean, D_{FROC}-mean, β_{FROC}-mean, and μ_{FROC} values were 1.446*10^{-3}mm^2/s, 1.517*10^{-3}mm^2/s, 0.864, and 9.623, respectively.

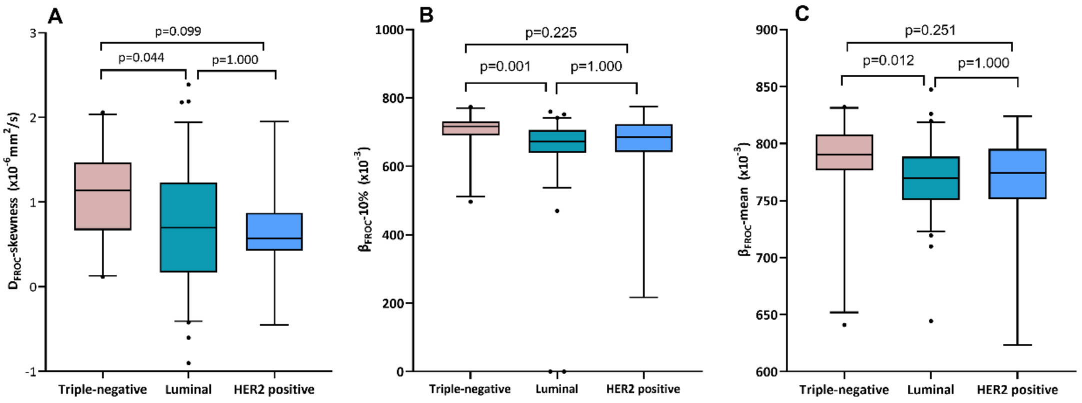

Supplementary Figure 3. Boxplots of (A) D_{FROC}-skewness, (B) β_{FROC}-10%, and (C) β_{FROC}-mean values. The top and bottom lines of the box represent the 5th–95th percentile values, and the line in the box represents the median value. D_{FROC}-skewness differed significantly between the triple-negative and HER2-positive, triple-negative and Luminal. β_{FROC}-10% and β_{FROC}-mean values showed significant

difference between the triple-negative and HER2-positive, triple-negative and Luminal.
